# Prevalence and Recognition of Cardiovascular Risk Factors in 308 Women Consulting Their GP for Any Reason

**DOI:** 10.1177/26884844251383337

**Published:** 2025-09-26

**Authors:** Roxanne Maghbouleh, Matthieu Calafiore, Matei-Eduard Pretorian, Sophie Catteau-Jonard, Marc Bayen, Nassir Messaadi, Sabine Bayen

**Affiliations:** ^1^ District of General Practice, University of Lille, Lille, France.; ^2^ MSPU Wattrelos, University of Lille, Lille, France.; ^3^ METRICS, University of Lille, Lille, France.; ^4^ Cardiology Practice, Roost-Warendin, France.; ^5^ Department of Medical Gynaecology, Orthogeny, Sexology, University of Lille, CHU Lille, University of Lille, Lille, France.; ^6^ MSPU Guesnain, University of Lille, Lille, France.; ^7^ PSPU Lille, University of Lille, Lille, France.; ^8^ Lille Neuroscience & Cognition U1172, Univ. Lille, Inserm, CHU de Lille, University of Lille, Lille, France.

**Keywords:** awareness, cardiovascular health, primary care, risk factors, women

## Abstract

**Introduction:**

Cardiovascular disease remains the leading cause of death among women. Across the lifespan, exposure to both traditional and lifestyle-related cardiovascular risk factors (CVRFs) is high. Behavioral trends such as increased sedentary behavior, psychosocial stress, and tobacco use have diminished the protective cardiovascular effects classically attributed to estrogen, with women underestimating their personal CVRF.

**Objective:**

To assess the prevalence and recognition of CVRF among women over 18 years attending general practice in the north of France.

**Materials and Methods:**

An exploratory, cross-sectional study was conducted between October 2023 and June 2024 in 10 general practices. Women aged ≥18 or older completed a self-administered online questionnaire, which assessed 17 established CVRF and their recognition of these as risks.

**Results:**

Among the 308 participants, the prevalence of CVRF was perceived psychosocial stress 69%, sedentary lifestyle 56%, overweight (body mass index >25) 36%, abdominal obesity (waist circumference >88 cm) 26%, hypertension 13%, diabetes 5%, and hyperlipidemia (low-density lipoprotein >1.6 g/L) 11%. Most (91%) reported at least two modifiable CVRF; 74% had at least three. Prevalence estimates for hypertension, diabetes, and hyperlipidemia increased sharply with age. The majority recognized smoking (86%), overweight (61%), and hypertension (83%) as risks, but only 29% recognized menopause and 57% identified estrogen-containing contraception as such.

**Conclusions:**

A substantial proportion of women, including the younger age groups, exhibit multiple coexisting modifiable CVRF, underscoring the need for early, targeted prevention. General recognition is high for some CVRF, but knowledge about those specific to women remains insufficient. Broad lifestyle determinants must be considered for universal CVRF prevention and management in primary care.

## Introduction

Cardiovascular disease (CVD) is the leading cause of death worldwide.^1^ In the member countries of the European Society of Cardiology, CVD is responsible for 45% of deaths among women, compared with 39% among men, revealing an underestimated disparity.^2^

In France, in 2022, almost 75,000 women died because of CVD, that is, around 200 deaths a day.^3^ The prevalence and impact of cardiovascular risk factors (CVRFs) differ between men and women.^4,5^ Even young, non-menopausal women may have specific hormonal risks associated with contraception, endometriosis, polycystic ovary syndrome, gestational diabetes, preeclampsia, miscarriage, age at first menarche, hysterectomy, and menopause. Hypertension, smoking, stress, and diabetes have more serious arterial consequences in women than in men.

The change in young women’s lifestyles, which are increasingly characterized by smoking, a sedentary lifestyle, and psychosocial stress,^6^ contributes to reducing the cardiovascular protection traditionally attributed to estrogen impregnation.^7^

The INTERHEART study showed that the risk of heart attack linked to smoking, high blood pressure, diabetes, or stress is higher in women than in men.^8,9^ These CVRFs are often less well detected in women than in men.^10^ Scores exist for assessing and predicting cardiovascular risk (CVR): the SCORE-2^11^ and the Framingham score.^12,13^ They distinguish between the two sexes, but do not consider female-specific CVRF. Regarding the age of screening, the Framingham score^13^ can be used from the age of 30, the SCORE-2^11^ from the age of 40, and the Reynolds score,^14^ which is female specific, from the age of 45.

In France, the French Society of Arterial Hypertension has proposed a CVR stratification for women in 2019, incorporating hormonal, obstetric, and behavioral CVRF.^10^

Nevertheless, to date, there is no score to assess the specific CVR of women younger than 30, and the prevalence of CVRF alone or in combination remains under-researched.

In 2022, the American Heart Association (AHA)^15^ alerted the scientific and health care communities and called for risk calculators that incorporate quantitative measures of risk in women to be used throughout life, particularly during follow-up in primary care.

Recent AHA guidelines identify seven key parameters for cardiovascular health (smoking, body mass index [BMI] >25 kg/m^2^, physical activity, diet, cholesterol, hypertension, diabetes) as priorities for primary prevention. Some CVRFs such as psychosocial stress, sedentary behavior, and overweight are increasingly ubiquitous, particularly among young women. In addition, women experience unique hormonal and reproductive CVRF. Current risk assessment tools may not adequately account for these early or overlapping risks, especially among women under age 30, contributing to a gap in both targeted prevention and research.

These parameters can be easily and regularly assessed by the GP (nonsmoker, BMI <25 kg/m^2^, physical activity in line with objectives, diet in line with current recommendations, untreated total cholesterol <200 mg/dL, untreated blood pressure <120/<80 mm Hg, and fasting blood glucose <100 mg/dL). These parameters have been shown to be poor in young American women (aged 20–44) in general and during pregnancy in particular.^15^ The same finding was made in 2022 in France among 2000 young women aged between 16 and 45.^16^ Of these, 73.8% had at least one CVRF. In terms of modifiable CVRF, 31% of the women were overweight or obese, 24% smoked tobacco or cannabis, 55% were sedentary, 84% ate little fruit and vegetables, and 87% had a low level of health literacy. In terms of cumulative CVRF, 73% had at least two traditional CVRF combined with at least one modifiable risk factor (RF). Regarding combined hormonal contraception, 34.0% of women presented one or more CVRF contraindicating their prescription according to the recommendations.

Concerning the diagnosis of an acute cardiovascular event, this may be delayed if women tend to minimize their symptoms, prioritize the health of those around them before their own,^17^ or when they do not recognize their CVRF or the symptoms of an acute cardiovascular event.^17^ Possible atypical symptoms of an acute coronary event in a woman can also mislead health care professionals.^18,19^ The atypical symptoms may be present as back, interscapular, epigastric, or mandibular pain or dyspnea.^20^

It is essential to improve the screening, identification, and management of CVRF in women throughout their lives.^5,15^

Moreover, improving individual recognition of CVRF is now considered a pivotal lever for engaging women in preventive care, yet data on awareness, especially in young women, remain scarce. In primary care, general practitioners (GPs) are strategically positioned to assess, inform, and motivate women regarding cardiovascular prevention.

It is therefore interesting and relevant to look at the potential CVR profiles of women aged 18 and over consulting their GP for any reason. To address this, we aimed to characterize the prevalence and overlap of a comprehensive range of CVRF (of both lifestyle/behavioral and traditional medical risks), extending beyond the AHA-defined risks, and to assess how well women recognize these risks in themselves.

## Materials and Methods

### Study design

A cross-sectional quantitative descriptive study was carried out in 10 GP practices in rural and urban areas in the north of France between October 2023 and June 2024. An anonymous, standardized questionnaire accessible online (LimeSurvey® software) was offered to women *via* a quick-response (QR) code in the waiting room and in the consulting room of the 24 GPs taking part in the study. Each GP was instructed to systematically ask each woman aged 18 and over to complete the brief online self-questionnaire. The women could complete the questionnaire either in the waiting room or during a consultation. Responses were collected anonymously. The GP was allowed to help fill in the questionnaire if necessary.

### Population studied

The inclusion criteria were women aged 18 or over, living in the north of France, consulting their GP for any reason, and agreeing to take part in the study.

### Questionnaire

The questionnaire used in this study consisted of 17 closed questions representing the main CVRF. The questionnaire is available in the Supplementary Data. It was designed based on cardiovascular prevention recommendations, particularly those targeting the female population.^12,13,15,21^

The non-modifiable CVRFs considered were age, heredity, and menopause. Modifiable CVRFs included contraception containing estrogen, arterial hypertension, diabetes, low-density lipoprotein (LDL) >1.6 g/L, high-density lipoprotein (HDL) <0.4 g/L, overweight, waist circumference (WC) >88 cm, lack of physical activity, sedentary lifestyle, psychosocial stress, high salt intake, smoking, and alcohol consumption.

At the end of the questionnaire, the women’s recognition of these RF was assessed using a single multiple-choice item, asking which listed variables participants considered CVRF (Supplementary Data).

This last part merely assessed whether women recognize the listed variables as potential CVRF and does not reflect or assess whether they possess true awareness or understanding.

### Data collection

The responses were automatically centralized online by the LimeSurvey software.

### Data analysis

The data were analyzed by age group (5 years between 18 and 50, and women over 50). First, a univariate descriptive analysis was carried out to characterize the prevalence of each CVRF, overall and by age group, followed by an analysis of their recognition of them for each group. Then, a co-occurrence analysis (prevalence of individuals with ≥2 and ≥3 RFs, both total and by subgroup) and a sensitivity analysis (addressing redundancy between related factors [*e.g.*, BMI and waist, sedentary and insufficient physical activity]) were provided by counting only one representative in each “covarying” pair.

The results were expressed as absolute frequencies and percentages. To assess a statistically significant association, a chi-square test was performed according to age group, for each variable, with a significance threshold set at *p* < 0.05.

An awareness/recognition analysis was conducted to assess the proportion recognizing each variable as a CVRF.

### Authorization

In the interests of data protection and personal freedom, the questionnaire (Supplementary Data) was validated by the Lille University Data Protection Department on May 31, 2023, and exempted from further declarations. All responses were centrally and anonymously collected.

## Results

### Participant characteristics

The study included 308 women 18 and over. They were divided into two groups: the first aged 18–50 and then subdivided into age groups (18–24, 25–29, 30–34, 35–39, 40–44, and 45–49) and the second aged 50 and over, due to the significant increase in CVRF after this age, as suggested by recommendations and data in the literature.

The following Table 1 shows the breakdown of women by age group.

**Table 1.** Breakdown of Participants by Age Group

Age range (years)	Quantity (*n* = 308)	Proportion (%)
18–24	26	8
25–29	103	33
30–34	46	15
35–39	24	8
40–44	24	8
45–50	17	6
>50	68	22

### Prevalence of individual and frequent CVRF

**FIG. 1.**
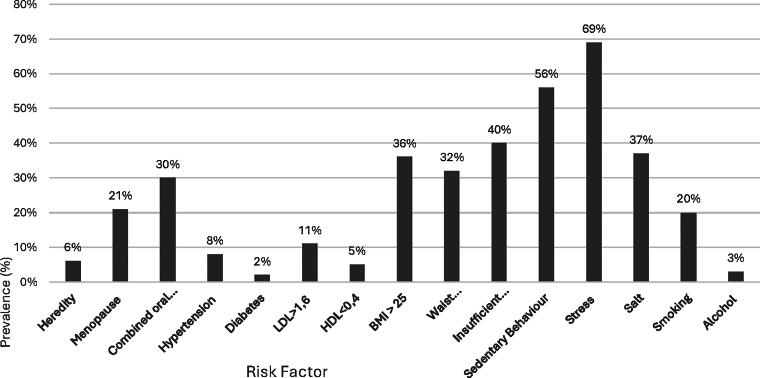
Prevalence of cardiovascular risk factors.

The following Figure 1 shows the prevalence of CVRF in the sample.

**FIG. 2.**
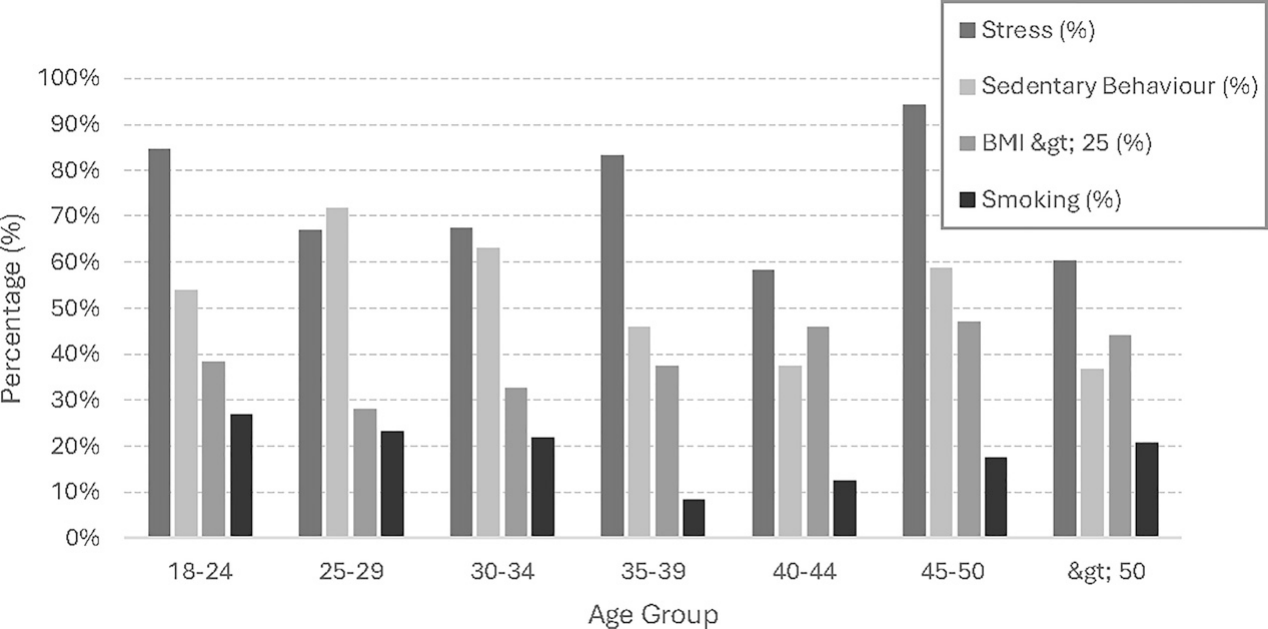
Prevalence of frequent risk factors by age group.

The following Figure 2 shows the prevalence of frequent CVRF by age group.

Among the CVRF studied, stress, a sedentary lifestyle, and BMI predominate. Participants were free to define stress as they wished, which could include minimal/light stress.

Sixty-nine percent of women (*n* = 213) self-declared to feel stressed. Stress was significantly higher among women aged 45–50 (94%; *p* = 0.027), compared with other age groups. Among women aged 18–24, 85% also reported stress.

More than half of the women declared to be sedentary (56%, *n* = 172), with a high prevalence in the 25–29 age group (72%), significantly so (*p* ≤ 0.01). This trend is confirmed in older age groups, particularly among women aged 45–50 (59%). Nearly half of these sedentary women do not engage in regular physical activity.

One hundred and twelve women in the sample (36%) declared a BMI >25. The majority (71%) combine this RF with abdominal obesity (WC >88 cm). Abdominal obesity is more common in premenopausal women aged between 45 and 50 (*p* < 0.05).

Smoking was reported by 20% of the women (*n* = 63) and concerned all age groups: 18–24 (27%), 25–29 (23%), and over 50 (21%).

Regarding diet-related CVRF, 37% of the women in the sample (*n* = 115) declared to consume more salt than recommended.^13^ Of these, 71% and 73% of the total sample were aware of salt as a RF.

An LDL cholesterol level >1.6 g/L was reported by 11% (*n* = 33) of women, with a significantly higher prevalence in the over 50s (35%) (*p* = 0.01) compared to the other age groups. Sixty-six women (21%) indicated that they did not know their LDL level.

About HDL cholesterol, 5% of women reported a level of <0.4 g/L, but a significant proportion of participants (45%, *n* = 139) did not know their HDL level.

**FIG. 3.**
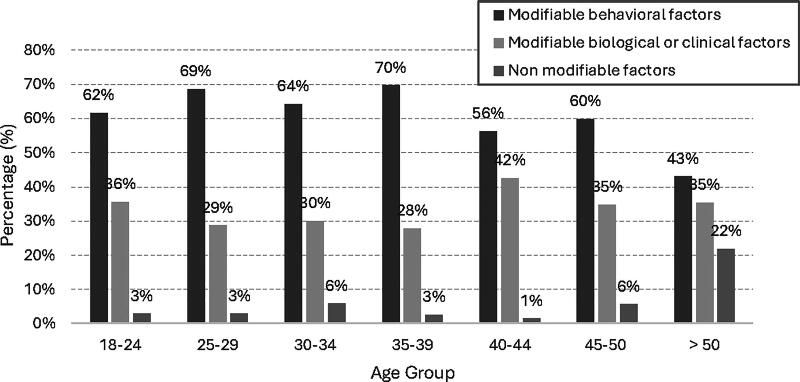
Categorical analysis of cardiovascular risk factors by group.

The following Figure 3 shows the categorical analysis of CVRF by age group.

### Overlap, co-occurrence, and sensitivity analysis of CVRF

Most women (91%) declared at least two CVRF, and 74% declared three or more. Among women aged over 50, 97% declared at least two RF, and 85% declared at least three.

Regarding overlap of CVRF among the 172 declared sedentary women (56%), 45% (*n* = 77) further declared being physically inactive, and 72% (*n* = 124) declared also stressed.

Among the 26 women of the sample who declared hypertension (8%), 17 (65%) declared to be stressed.

As for being overweight or obese, 73% of the women concerned (*n* = 82) declared to be stressed. Of the 63 women who smoke, 73% (*n* = 46) also report stress.

The following Table 2 shows the prevalence of CVRF of all kinds by age group.

**Table 2.** Prevalence of Cardiovascular Risk Factors by Age Group

Age (years)	At least two risk factors	At least three risk factors
18–24	85% (*n* = 22)	69% (*n* = 18)
25–29	92% (*n* = 95)	62% (*n* = 64)
30–34	91% (*n* = 42)	67% (*n* = 31)
35–39	92% (*n* = 22)	62% (*n* = 15)
40–44	75% (*n* = 18)	54% (*n* = 13)
45–50	94% (*n* = 16)	71% (*n* = 12)
>50	97% (*n* = 66)	85% (*n* = 58)
Total	91% (*n* = 281)	69% (*n* = 211)

We conducted a sensitivity analysis to address the issue of overlapping RF, selecting just one of the covarying factors as an indicator where appropriate to consider the importance of risk clustering, especially for prevalent lifestyle risks.

The following Table 3 shows the prevalence of detailed CVRF by age group.

**Table 3.** Prevalence of Detailed Cardiovascular Risk Factors by Age Group

Risk factor	Overall prévalence	Age trend
Psychosocial stress	69% (*n* = 213)	45–50 (94%, *n* = 16), *p* < 0.05
Sedentary lifestyle	56% (*n* = 172)	25–29 (72%, *n* = 74), *p* < 0.05
Physical inactivity	40% (*n* = 124)	30–34 (50%, *n* = 23) 35–39 (50%, *n* = 12)
Sedentary lifestyle and physical inactivity	25% (*n* = 77)	30–34 (37%, *n* = 17)
Sedentary lifestyle and/or physical inactivity	71% (*n* = 219)	25–29 (77%, *n* = 79)
BMI >25	36% (*n* = 112)	45–50 (47%, *n* = 8)
Abdominal obesity	32% (*n* = 98)	45–50 (53%, *n* = 9), *p* < 0.05
Both BMI and WC >88 cm	26% (*n* = 79)	45–50 (47%, *n* = 8)
BMI >25 and/or WC >88 cm	43% (*n* = 131)	40–44 (58%, *n* = 14)
Smoking	20% (*n* = 63)	18–24 (27%, *n* = 7)
Hypertension	8% (*n* = 26)	>50 (29%, *n* = 20), *p* < 0.05
Diabetes	2% (*n* = 6)	40–44 (8%, *n* = 2), *p* < 0.05 >50 (4%, *n* = 3), *p* < 0.05
LDL >1.6 g/L	11% (*n* = 33)	>50 (35%, *n* = 24), *p* < 0.05
HDL <0.4 g/L	5% (*n* = 15)	Many unknown values
Excess salt consumption	37% (*n* = 115)	25–29 (44%, *n* = 45)
Alcohol consumption	3% (*n* = 10)	30–34 (9%, *n* = 4)
Estro-progesterone contraception	30% (*n* = 91)	18–24 (58%, *n* = 15), *p* < 0.05
Menopause	21% (*n* = 65)	>50 (93%, *n* = 63) 45–50 (12%, *n* = 2)

Only significant results were followed by the p-value.

BMI, body mass index; HDL, high-density lipoprotein; LDL, low-density lipoprotein; WC, waist circumference.

### Awareness and recognition of CVRF

#### Specific CVRF known to women

In this sample, certain RFs, linked to specific conditions such as contraception or menopause, are known approximately according to age. Their prevalence in the sample varies according to age and behavior.

The use of estrogen–progestogen contraception is most common among young women, with 58% of 18- to 24-year-olds using it. Of the 91 women using estrogen–progestogen contraception, 63% (*n* = 57) recognized that it was a CVRF.

Nearly one in five women taking estrogen–progestogen contraception declared themselves smokers. Of these women (*n* = 18), 44% did not recognize that contraception containing estrogen represents a risk.

Twenty-nine percent of women recognized the menopause to be a CVRF, including 37% of women over 50.

**FIG. 4.**
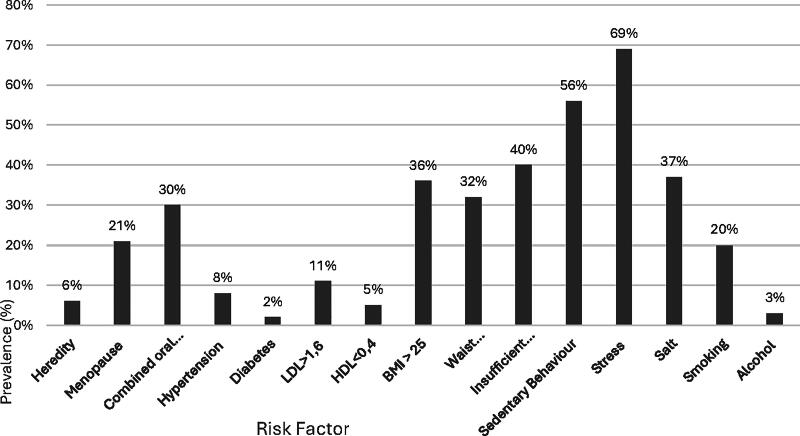
Prevalence of cardiovascular risk factors.

Figure 4 shows the prevalence, awareness, and recognition of specific RF.

### Recognition of the risks and behaviors observed

Notably, even among women who possessed an RF, individual perception of increased risk appeared limited.

Eighty-six percent of women recognized smoking as a CVRF, and 89% of women declared to smoke.

Sixty-one percent of overweight women recognized being overweight as a risk. Fifty-five percent of women with a BMI over 25 are also sedentary, and 47% do not engage in regular physical activity.

Half of the women in the sample recognized high LDL cholesterol as a CVRF.

Twenty-nine percent of women (*n* = 90) identified low HDL cholesterol as a CVRF.

**FIG. 5.**
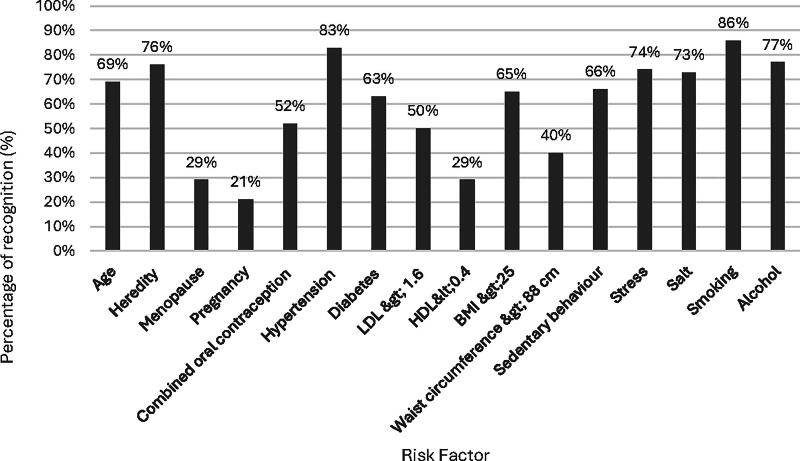
Recognition of cardiovascular risk factors.

The following Figure 5 shows awareness of CVRF in the sample.

## Discussion

This study demonstrates a high prevalence, from young adulthood onward, of modifiable CVRF including stress, sedentary behavior, and overweight among women attending general practice in northern France. Our results align with previous North American^15^ and French^16^ data yet provide heightened granularity for the context of primary care and the under 30 age group.

Three CVR profiles emerge according to age.

Women aged 18–35 declare more likely to be stressed, sedentary, and use the contraceptive pill. Between the ages of 35 and 50, stress and a sedentary lifestyle are compounded by excess weight, which is predominant in this population. After the age of 50, the combination of stress, excess weight, and menopause constitutes a potentially high-risk profile.

It is important to distinguish between a sedentary lifestyle and a lack of physical activity. A sedentary lifestyle involves prolonged periods of self-reported inactivity most of the day (sitting or lying down in a context of work or leisure) regardless of reported physical activity levels.^22^ Lack of physical activity refers to the absence of moderate or intense physical effort.^22^ A person can be both physically inactive and sedentary. When these two behaviors are combined, they add up and worsen CVR by contributing to high blood pressure, overweight, dyslipidemia, a chronic inflammatory state, and insulin resistance.^22–24^ Lack of regular physical activity could be responsible for around 12.2% of myocardial infarctions,^25^ which underlines the importance of acting on these two levers.

In young women, being overweight is associated with a significantly increased risk of myocardial infarction, coronary heart disease, and stroke, with events occurring before the age of 50. The higher the body mass index, the greater the risk.^16,26^

Self-reported stress emerges as the most predominant modifiable CVRF in this study, affecting women of all ages. This underlines the need to address this issue comprehensively in cardiovascular prevention strategies.

The INTERSTROKE study highlighted the link between different types of stress (financial, domestic, work-related) and an increased risk of stroke, whether ischemic or hemorrhagic.^27^ This risk is of particular concern in women, not only because of their more frequent exposure to sources of stress but also because stress has a greater cardiovascular impact in women.^28,29^ Sources of stress for women include socioeconomic inequalities, traumatic childhood events, domestic violence, marital experience, and caring responsibilities, bearing in mind that 80% of carers worldwide are women.^28^

In this study, a trend was observed between self-declared psychosocial stress^6^ and other CVRF such as hypertension or being overweight. Chronic stress also increases atherosclerosis and leads to endothelial dysfunction *via* inflammatory, neurohormonal, and metabolic mechanisms.^30,31^ In addition to its physiological effects, stress is often associated with risky behaviors that can worsen CVR. Faced with chronic stress, some women develop compensatory behaviors such as overconsumption of sugar-rich or high-calorie foods, increasing the risk of overweight or obesity.^32,33^ Others may increase their tobacco consumption or adopt a more sedentary lifestyle.^28^ The effects of stress on cardiovascular health are not limited to chronic stress. Acute stress can also have serious consequences, such as tako-tsubo syndrome, triggered by an intense emotional event and more common in women.^34^

### Recognition of CVRF

CVRF such as age, heredity, smoking or alcohol consumption, high blood pressure, diabetes, being overweight, or stress are mostly known. This recognition can be attributed to prevention campaigns that have been in place for several years or to more targeted awareness campaigns.

Findings suggest gaps, especially in awareness of “female” factors and lifestyle determinants.

There is a lack of information about CVRF specific to women, such as the menopause.

Diet-related CVRFs, in particular excessive salt consumption and high LDL cholesterol levels, were generally well-known. Little was known about low levels of HDL cholesterol as a CVRF. On the contrary, individual lipid profiles were less well identified, highlighting a need for optimization in the communication of biological results to women monitored by their GP. It should be noted that the study did not include triglyceride values, despite their important role in the risk of atheromatous plaque formation.^35^

It is interesting to note that despite their recognition of CVRF, more than half of women declare harmful behaviors, in particular a sedentary lifestyle that encourages excess weight. This may be influenced by factors such as stress, addiction, or the increased use of digital tools (television, telephone, computer), which indirectly reduce opportunities for physical activity. The sedentary nature of many professions and the lack of time are also frequently cited obstacles.^36^

Two studies carried out in the United States have shown that, although women are generally aware of CVRF, they have difficulty in seeing themselves as being concerned, revealing a deficit in individual risk acceptance.^37,38^

In addition to the CVRF examined in our study, there are other women specific CVRF which must be considered in primary care settings. These include early menstruation, polycystic ovary syndrome, infertility, other events related to pregnancy or childbirth (premature delivery, low or high birth weight), and early menopause.^15,39^ Autoimmune diseases such as lupus or rheumatoid arthritis also increase CVR through the chronic inflammation they cause.^40^ Certain treatments such as radiotherapy for breast cancer (particularly left breast)^41^ and treatments with anthracyclines or trastuzumab increase CVR.^20^ Conversely, certain protective factors such as breastfeeding are recognized for their benefits on cardiovascular health.^21,42^

All these CVRFs specific to women and the seven parameters for primary cardiovascular prevention^15^ can be easily assessed by GPs during consultations for any reason.

### Strengths and Limitations

Data on non-menopausal women treated in general practice are both necessary and rare. This study has several strengths.

We included women from the age of 18, which enabled us to paint a picture of the prevalence and recognition of CVRF in women before and after the menopause.

By assessing both recognition of RF and health behaviors, the study adopts a preventive approach. This makes it possible to identify gaps in recognition that need to be addressed in targeted information campaigns aimed at different profiles of women (from teenagers to postmenopausal women).

We intentionally elected to characterize a broad spectrum of CVRF including lifestyle and female-specific determinants, in addition to the AHA’s seven primary factors to better reflect the real-world landscape encountered in primary care and women’s health. While such inclusivity can dilute the “weight” of individual classical risks, it more accurately depicts cumulative risk environments and targets universal prevention, especially valuable in young women, who frequently present classical RF “clusters” rather than isolated medical risk.

Our study has limitations. The study is limited by its sample distribution (overrepresentation of 25–29 age group), potential self-report bias (especially for subjective and laboratory-based variables), and possible selection bias linked to GP-attending women and regional socioeconomic context.

The questionnaire (Supplementary Data), accessible *via* a QR code, spontaneously appealed to young women, who filled it in very quickly on their smartphones. The recruitment of participants from general practices could present a selection bias, as women who regularly consult their GP are potentially more involved in their health than the general population. Another selection bias is associated with recruiting women from the north of France, which has a higher proportion of population with increased structural socioeconomic vulnerability and a higher prevalence of CVRF and CVD than other regions of France.^43,44^ The self-reported nature of the data may introduce a social desirability bias, with women overestimating or underestimating their recognition. The assessment of stress could be biased because of our subjective and binary measurement. With the aim of offering a short, rapid questionnaire in general practices, we did not use the Perceived Stress Scale^45^ because it consists of 10 questions. To foster women’s participation, we just asked the simple question: “*Do you consider yourself to be ‘stressed’ at present (work, domestic, financial stress, recent distressing event, etc.)?*”

Nielsen^46^ used this question to grade the perceived stress level (none, light, moderate, high [0–3]). We can consider that all women of our study sample who answered with “yes” perceive at least a “light” stress level.^46^

While this may overestimate the prevalence of clinically significant stress, it captures an important subjective risk environment. Literature suggests that even mild perceived stress can contribute to cardiovascular pathology through behavioral and neurohumoral mechanisms.

The same applies to physical activity, which we answered in a single binary question. Despite this, we can consider that the women who answered “no” were at best active for 1 hour 15 minutes and at worst less, while those who answered “yes” were at least active for 1 hour 15 minutes and at best active for more than 2 and a half hours a week.

Again, regarding sedentary behavior, we must consider that it was likewise measured by a single question that could potentially classify all women in administrative jobs as having a sedentary lifestyle.

Some women who completed the questionnaire (Supplementary Data) on their own, without the assistance of a doctor, may have underestimated certain CVRF, particularly in relation to biological parameters.

A chi-square test was performed for each of the variables. This may have led to alpha risk inflation bias due to the multiplicity of comparisons.

The grouping of overlapping co-occurring CVRF likely inflated the proportion with two or more risks, but our sensitivity analysis addressed this limitation.

The high frequency and overlap of certain modifiable lifestyle CVRF such as psychosocial stress and sedentary behavior means that the rate of women with two or more CVRF is considerable but may not directly equate to higher clinical risk in all cases. For instance, mild self-reported stress or a BMI just >25 kg/m^2^ may confer limited additional risk compared to more severe, persistent factors (references). Given the design of our questionnaire (Supplementary Data), we could not assess stress severity or BMI extremes; future studies should incorporate validated instruments and distinguish between RF grades.

Finally, the use of a more “comprehensive” set of RF, as compared to AHA, makes direct comparison with some published literature challenging.

## Conclusions

Our findings reveal that even among very young women, the burden of cumulative modifiable CVRF is substantial, driven partly by overlapping lifestyle and behavioral factors. While awareness of classical CVRF remains high, knowledge concerning female-specific and lifestyle determinants is uneven. Comprehensive, early, and tailored preventive actions, initiated in primary care, are essential. Approaches integrating the full spectrum of CVRF beyond the AHA’s seven may more effectively target the actual needs of women across the lifespan.

## Supplementary Material

Supplementary Data

## Abbreviations Used

BMI: body mass index
CVD: cardiovascular disease
CVR: cardiovascular risk
CVRF: cardiovascular risk factors
GP: general practitioner
HDL: high-density lipoprotein
LDL: low-density lipoprotein
RF: risk factor
WC: waist circumference
