# Supplementary material for: Prevalence and Recognition of Cardiovascular Risk Factors in 308 Women Consulting Their GP for Any Reason
Source: Womens Health Rep (New Rochelle). 2025 Sep 26;6(1):1034–44. doi: 10.1177/26884844251383337 (PMC12549180; doi:10.1177/26884844251383337)
Supplement: Supplementary Data [file 26884844251383337_suppl_data.docx]

**Questionnaire**

**1-How old are you?**

o 18-24 years old

o 25-29 years old

o 30-34 years old

o 35-39 years old

o 40-44 years old

o 45-50 years

o >50 years

**2- Do you have a first-degree relative (mother or father) who had a myocardial infarction (heart attack) before the age of 55 (if male) or before the age of 65 (if female) or a stroke before the age of 45?**

o Yes

o No

o Don't know

**3- Are you post-menopausal?**

o Yes

o No

**4- Are you taking contraception containing oestrogen (oestroprogestogenic pill, patch or ring)?**

o Yes

o No

**5- Is your waist greater than 88 cm? (measure with your doctor if necessary)**

o Yes

o No

**6- Is your weekly physical activity**

less than 2 hours of moderate activity (walking, housework, gardening, leisure cycling, dancing, golf, etc.) or less than 1 hour of sustained activity (brisk walking, hiking, ball games, tennis, etc.)?

ball games, tennis...)

o Yes

o No

**7- Do you spend more than 6 hours sitting (in front of a**

**a computer or television) in a day?**

o Yes

o No

**8- Do you consider yourself to be ‘stressed’ at present (work, domestic, financial stress, recent distressing event, etc.)?**

o Yes

o No

**9- Do you have high blood pressure? (BP > 140/70 mmHg) (If you are taking treatment for hypertension, please tick yes)**

o Yes

o No

**10- Do you have diabetes?**

o Yes

o No

**11- Do you have hypercholesterolaemia (‘high cholesterol’)? (LDL > 1.6 g/L) (if you do not have a recent blood test, tick ‘don't know’)**

o Yes

o No

o Don't know

**12- Do you have HDL cholesterol < 0.4 g/L? (if you do not have a recent blood test, tick don't know)**

o Yes

o No

o Don't know

**13 - Do you have a BMI > 25?**

o Yes

o No

**14- Do you eat more than 6g of salt a day? (for information, there is 1g of salt in a slice of sausage, a handful of crisps, an aperitif biscuit, four slices of bread, a slice of pizza, a third of a ham sandwich...)**

o Yes

o No

**15- Do you smoke?**

o Yes

o No

**16- Do you drink more than one glass of alcohol a day?**

o Yes

o No

**17- All the above are cardiovascular risk factors. Which of these did you know?**

o Age

o Heredity

o Menopause

o Contraception with oestrogens

o Hypertension

o Diabetes

o High LDL

o Low HDL

o BMI > 25

o Waist > 88 cm

o Sedentary lifestyle

o Stress **(psychosocial: work, domestic, financial stress, recent distressing event, etc)**

o High salt intake

o Smoking

o Alcohol
